# The role of water in protein's behavior: The two dynamical crossovers studied by NMR and FTIR techniques

**DOI:** 10.1016/j.csbj.2014.11.007

**Published:** 2014-11-15

**Authors:** Francesco Mallamace, Carmelo Corsaro, Domenico Mallamace, Sebastiano Vasi, Cirino Vasi, Giacomo Dugo

**Affiliations:** a Dipartimento di Fisica e Scienze della Terra, Università di Messina, Viale F. Stagno D'Alcontres 31, 98166 Messina, Italy; b Dipartimento di Scienze dell'Ambiente, della Sicurezza, del Territorio, degli Alimenti edella Salute, Università di Messina, Viale F. Stagno d'Alcontres 31, 98166 Messina, Italy; c CNR-IPCF, Istituto per i Processi Chimico-Fisici, Viale F. Stagno D'Alcontres 37, 98158 Messina, Italy

**Keywords:** Protein dynamic transition, Amide bending mode, Lysozyme unfolding, Hydration water, HR-MAS

## Abstract

The role the solvent plays in determining the biological activity of proteins is of primary importance. Water is the solvent of life and proteins need at least a water monolayer covering their surface in order to become biologically active. We study how the properties of water and the effect of its coupling with the hydrophilic moieties of proteins govern the regime of protein activity. In particular we follow, by means of Fourier Transform Infrared spectroscopy, the thermal evolution of the amide vibrational modes of hydrated lysozyme in the temperature interval 180 K < *T* < 350 K. In such a way we are able to observe the thermal limit of biological activity characterizing hydrated lysozyme. Finally we focus on the region of lysozyme thermal denaturation by following the evolution of the proton Nuclear Magnetic Resonance (NMR) spectra for 298 K < *T* < 366 K with the High-Resolution Magic Angle Spinning probe. Our data suggest that the hydrogen bond coupling between hydration water and protein hydrophilic groups is crucial in triggering the main mechanisms that define the enzymatic activity of proteins.

## 1 Introduction

Protein activity is connected with their hydration water [1]. In fact, at least, a monolayer of water molecules called the first hydration shell or directly hydration water, extended over the protein surface is needed for the execution of the enzymatic activity [2], [3]. The key factor of protein hydration is the H-bonding between protein surface polar groups and hydration water. Furthermore, the coupling between the hydration water and the hydrophilic moieties of the protein surface triggers the search for the correct native state (protein folding): the complex heteropolymeric amino acid sequences spontaneously fold up into organized three-dimensional structures. The spontaneity of the folding process depends on the occurrence of non-functional, or promiscuous, interactions between any suitable pair of residues that can provoke the formation of transient intermediate structures, through “non-native” interactions or frustration [4], [5], [6], that can be interpreted as roughness on the folding energy landscape [7]. The native state of a protein corresponds to a global free energy minimum that the protein reaches in a time range from microseconds to seconds [8]. If the protein should find its native state just by random searching (Levinthal paradox [9]) among the huge number of possible conformations, this search could take longer than the age of the universe.

It is noteworthy that already in 1936, Mirsky and Pauling discriminated between the fundamentally different character of the native and denatured states of proteins [10]. They argued that the folding process is no more (nor less) miraculous than is the formation of a crystal from a supersaturated solution [10]. The reason lies in the cooperative nature of the denaturation and in the large magnitude of the corresponding enthalpy change. Indeed the native state must be nearly unique in structure, like a crystal, whereas the denatured state has a much higher entropy, reflecting the numerous disordered conformations that a chain molecule could take on [10]. However one has to consider that the protein is a finite system and the extension of concepts such as those of nucleation and growth mechanism cannot be easily applied. From a physical point of view, proteins are hard matter at low temperature, whereas they are soft matter at high temperature depending on the competition between the enthalpic end entropic contributions [8]. The highly directional and polar character of the hydrogen bond seems to be the key to understand the microscopic mechanisms occurring during protein folding [10], [11]. In fact, the physical and chemical properties of proteins depend on the characteristic of the hydrogen bonds formed within the protein residuals and with its hydration water.

For lysozyme in particular it has been shown how the hydrogen bond network that hydration water develops on the protein surface is stable at atmospheric pressure within a temperature interval going from ≈ 225 K to ≈ 320 K [12], [13], [14], [15], [16], [17], [18]. The temperature of 225 K has been identified as the temperature of the protein glass-transition but its nature is up to now the subject of many controversies [17], [18], [19], [20], [21], [22]. In fact, it was pointed out that the glass transition temperature of hydrated lysozyme depends on the hydration level, and the time scale of measurement [23], [24]. Below 225 K, the water hydrogen bond network is highly rigid being fully developed and protein side-chains motion is hindered. On the contrary, above 320 K the lifetime of the hydrogen bond is too short (less than picoseconds) and does not allow to keep together the protein residuals giving rise to the unfolding process [16], [25], [26], [27]. The native state of lysozyme does not evolve directly into the completely unfolded (or denatured) state but passes through an intermediate state (within which the unfolding process is reversible) where rapid conformational changes occur and can provoke alteration of the folding. The alteration of the folding (or misfolding) of proteins is the source of neurodegenerative illnesses such as Alzheimer's and Parkinson's diseases [7], [28].

In this paper we use two different but complementary techniques such as Fourier Transform Infrared (FTIR) and Nuclear Magnetic Resonance (NMR) spectroscopies to investigate how the coupling between the hydration water and the protein residuals evolves as a function of the temperature and determines the limits of biological activity of hydrated lysozyme. In particular, with FTIR we were able to probe the interval from 180K to 360K with a 10K step, whereas with NMR we focused on the unfolding process from 298K to 366K with a 2K step.

## 2 Materials and methods

Lysozyme is a small protein of 14.4 kDa; it is constituted by 129 amino acid residuals and in the native state has a globular shape. Lysozyme is easily found in animal tissues and displays anti-inflammatory and antibacterial properties. Protein samples were obtained from Fluka (L7651 three times crystallized, dialyzed, and lyophilized) and used without further purification. Samples were dried, hydrated isopiestically, and controlled by means of a precise procedure [12]. Our aim is to study the first monolayer of water molecules surrounding the protein surface and this corresponds to a hydration level, h (grams of water per gram of dry protein) equals to 0.3. Fourier Transform Infrared (FTIR) absorption measurements were performed by means of a Bomem DA8 Fourier transform spectrometer, operating with a Globar source, in combination with a KBr beamsplitter and a DTGS/KBr detector. We operated in the attenuated total reflection (ATR) geometry to avoid saturation effects. Spectra were recorded with a resolution of 4 cm^− 1^, automatically adding 200 repetitive scans in order to obtain a good signal-to-noise ratio and highly reproducible spectra; then they are normalized by taking into account the effective number of absorbers [13]. Measurements were performed at ambient pressure in the spectral region from 1300 cm^− 1^ to 1750 cm^− 1^, in the temperature range from 180 K to 350 K. Proton NMR experiments were performed at atmospheric pressure in the temperature range 298 K < *T* < 366 K by using a 700 MHz Bruker Avance spectrometer equipped with the Magic Angle Spinning (MAS) probehead. Hydrated protein samples were placed in a 50 μl rotor and spun at 4000 Hz at the magic angle to increase the spectral resolution. By tilting samples of a precise angle with respect to the applied magnetic field, the hamiltonian term corresponding to the dipolar interactions vanishes and NMR peaks become narrower. Furthermore, by spinning the rotor at the magic angle by few thousands of Hertz, line broadening effects due to susceptibility differences within the sample are removed resulting in high resolution quality spectra. The sample temperature was controlled by a cold N_2_ flow and a heating element, calibrated by using the frequency shift between ethylene glycol peaks. The duration of the hard pulse was 8 μs with a relative attenuation of 3 dB; the spectral width was 10 kHz, the acquisition time 2.9 s, the points in the time domain 64 k, the number of transient 128 and the relaxation time 2 s for a total time of about 10 min per experiment. All spectra were processed (line broadening, Fourier transform, phase correction and baseline adjustment), by using the standard routines of the Bruker software Xwinnmr version 3.5.

## 3 Results and discussions

InfraRed and NMR spectroscopies are probably the most used experimental techniques able to study protein structure and properties [29]. In particular, FTIR spectroscopy permits a detailed analysis of the structure and stability of proteins, using peptide backbone and side-chain marker bands as conformation-sensitive monitors. Specific information on the secondary structure of proteins is obtained from the analysis of the various amide bands which are indeed sensitive to the protein conformation.

In details, IR spectra of hydrated proteins provide useful structural information especially in the region of Amide I (the most intense band centered at 1600 − 1700 cm^− 1^) which is mostly a carbonyl (C=O) stretching [30], [31]. In particular, the amide I band is sensitive to hydrogen bond pattern, dipole–dipole interaction and the geometry of the polypeptide backbone. It consists of several overlapping bands of different structural elements that could be studied separately by means of a peak deconvolution [30], [32].

**Fig. 1:**
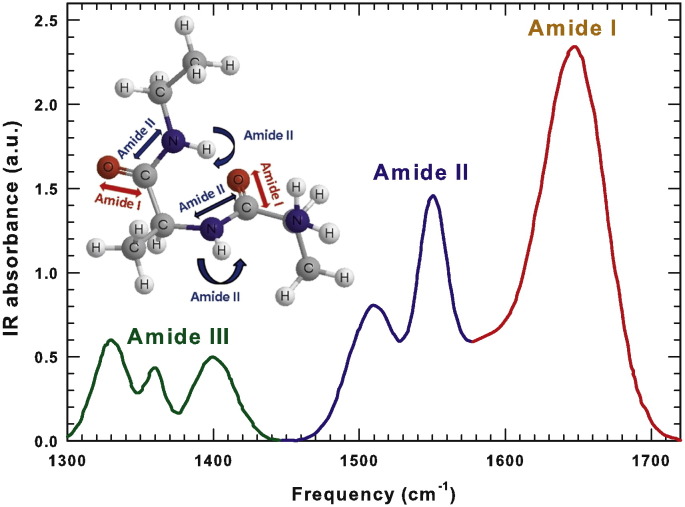
The different Amide contributions to the IR bending region are reported with different colors in the interval 1300 − 1720 cm^− 1^. The Amide I and II vibrational modes are also represented on a peptide fragment using the same color of the corresponding IR frequency regions.

Other intense and important Amide bands are Amide II and Amide III extending respectively from 1480 to 1580 cm^− 1^ and from 1300 to 1450 cm^− 1^. The Amide II mode is essentially the combination of the N–H in plane bending and of the C–N stretching, while Amide III consists of more complex vibrational modes [31], [33]. The different Amide contributions to the IR bending region are reported in Fig. 1 with different colors. In the figure, the Amide I and II vibrational modes are also represented on a peptide fragment using the same color of the corresponding IR frequency regions.

**Fig. 2:**
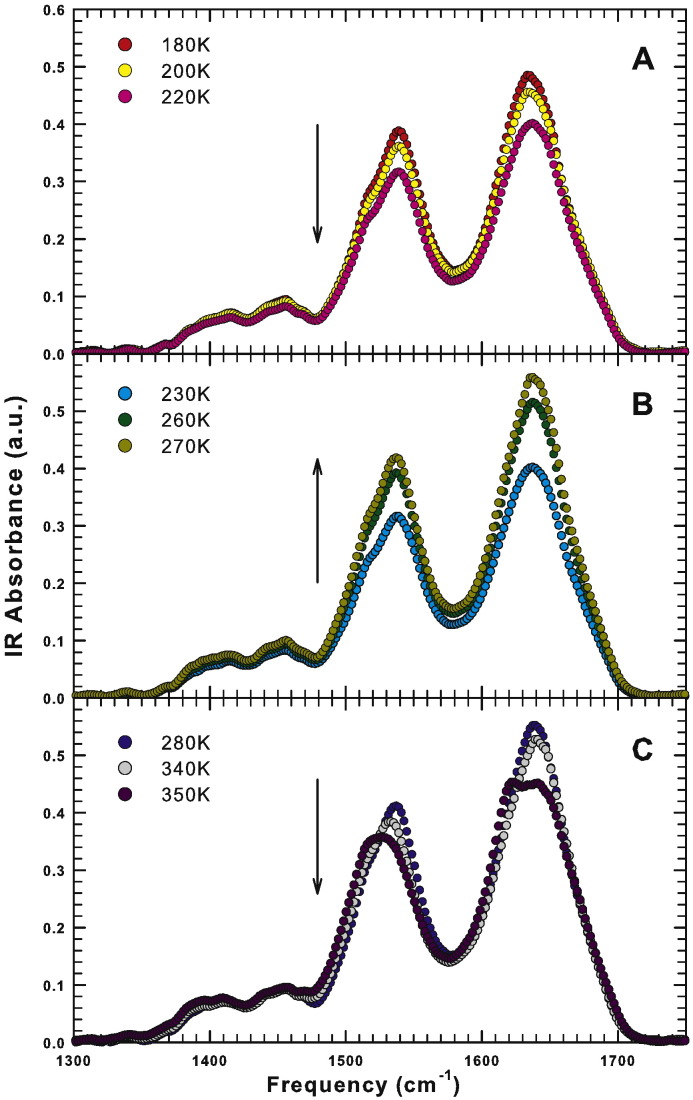
The Infrared spectra of hydrated lysozyme (h = 0.3) in the interval 1300 − 1750 cm^− 1^ for 180 K < *T* < 220 K (panel A), for 230 K < *T* < 270 K (panel B) and for 280 K < *T* < 350 K (panel C). The arrows indicate the evolution of the signal intensity with temperature.

The hydrogen bond coupling is a complex phenomenon that can be studied by analyzing the trend that IR spectra show as a function of the temperature. In particular, the behavior of the Amides peak intensity, on increasing the temperature in all the studied range, is not monotonic. In Fig. 2 we use three panels to separate the three important thermal regions within which the spectral behavior is monotonic with temperature. In each panel, for clarity we report only three significant temperatures able to describe the thermal behavior; the intermediate temperatures follow the same trend. In particular, panel A of Fig. 2 shows the IR spectra in the low temperature region from 180 K to 220 K. The signal intensity decreases on increasing the temperature as indicated by the arrow. Panel B of Fig. 2 shows the IR spectra in the intermediate temperature region, from 230 K to 270 K. Note that, in the intermediate temperature region, the signal intensity increases with the temperature. Panel C of Fig. 2 shows the IR spectra in the high temperature region, from 280 K to 350 K. Again, in this high temperature region, the signal intensity decreases on increasing the temperature. The shoulder at about 1500 cm^− 1^, associated with the amide II N–H residual, is well evident at low temperature and disappears upon denaturation [34].

**Fig. 3:**
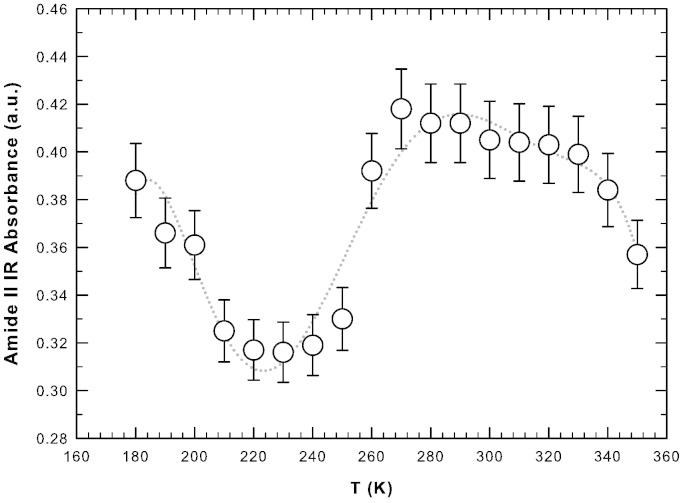
The intensity of the Amide II infrared band as a function of the temperature for hydrated lysozyme (h = 0.3). The dotted line is a polynomial best fit as a guide for the eye.

One suitable approach for the characterization of temperature-induced conformational changes in protein is to construct intensity/temperature profiles for selected IR bands. In such a way one can determine standard thermodynamic properties of the system such as transition or crossover temperatures [35]. Fig. 3 shows the intensity of the Amide II band as a function of the temperature in the considered thermal range. The dotted line is a polynomial best fit as a guide for the eye. The Amide II band, representing the N–H bending contribution, reflects directly the coupling between hydration water and protein residuals. Note that, on increasing the temperature, the intensity of the Amide II band shows a minimum at ≈ 225 K. This temperature coincides with that of the dynamical crossover observed by means of neutron scattering [12], [36] and NMR spectroscopy [13], [27], [37]. It is noteworthy that even though these experimental techniques encompass different time scales, all lead to the same temperature range for the occurrence of the dynamical transition. Above 225 K, when the motional amplitude (i.e., the flexibility) of hydrated lysozyme sharply increases, the intensity of the Amide II band increases with temperature up to ≈ 270 K. Then, the intensity of the Amide II band starts to slowly decrease on increasing the temperature with a smooth inflection point at ≈ 320 K. This is the “magic” temperature above which water behaves as a normal liquid since the HB lifetime becomes too small (less than picoseconds) and all water molecules are essentially free [38]. Furthermore at this temperature the refolding rate constant assumes the maximum value [8]. Above 320 K the intensity of the Amide II band sharply decreases on increasing the temperature (Fig. 3); water is no more a “good solvent” and lysozyme loses its globular structure evolving toward a linear chain of amino acids. Thus, two temperatures appear to be relevant for the onset of different dynamical regimes (and so to the functioning) of hydrated lysozyme. The values of these temperatures agree with those already found for the same system and described in the introduction, which are 225 K and 320 K. All the described changes and their thermal borders are well described by the intensity of the Amide II band reported in Fig. 3 confirming the ability of the FTIR technique in detecting the structural conformational behavior of proteins and the corresponding role of hydration water. The spectrum at 350 K, which is the highest measured temperature, shows (panel C) a clear peak bifurcation relatively to the Amide I band at 1650 cm^− 1^. This is due to the onset of aggregation processes in which *α* -helices transform into *β*-sheets that tend to self-aggregate. The unfolding process is reversible in character up to ≈ 346 K and then becomes irreversible [26].

**Fig. 4:**
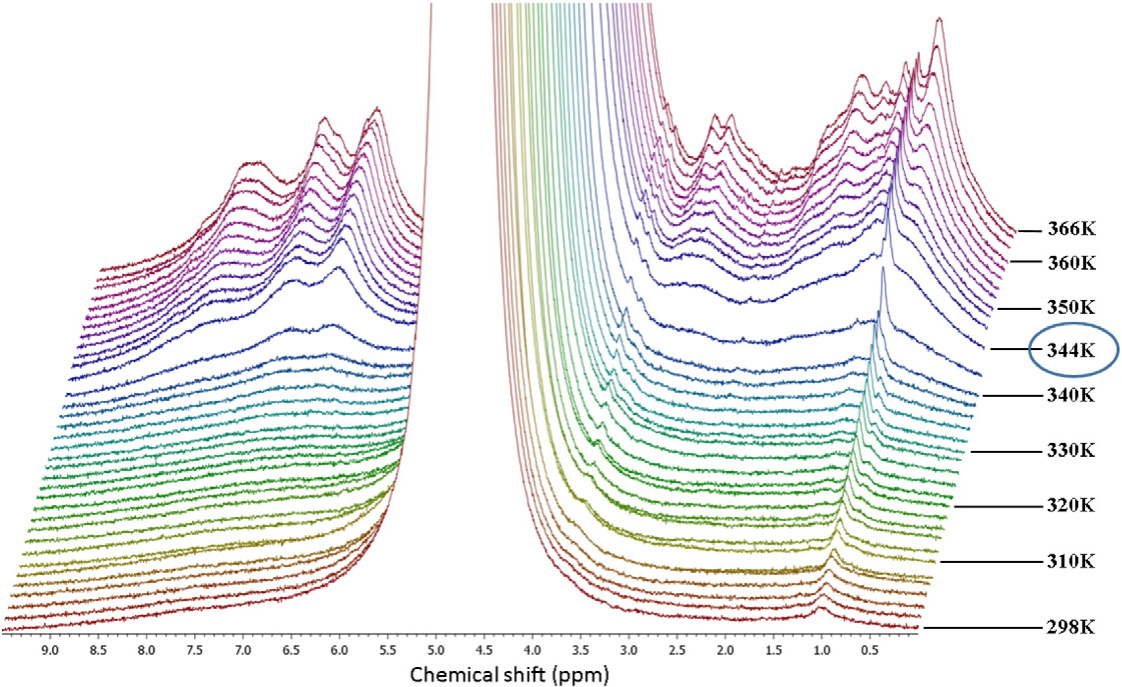
The stacked plot of NMR spectra for hydrated lysozyme (h = 0.3) in the high temperature region measured by means of the HR-MAS set-up. The temperature of 344 K is highlighted because it marks the irreversibility of the unfolding process.

We performed a detailed NMR experiment in order to get a precise insight into the thermal denaturation of hydrated lysozyme. In fact, as shown in Fig. 3, aside from the smooth inflection point at about 320 K, the thermal trend of the intensity of the Amide II band for 280 K < *T* < 330 K is quite flat. NMR spectra of hydrated proteins allow studying the different chemical groups of protein and water separately. In Fig. 4 we present the stacked plot of NMR spectra for hydrated lysozyme (h = 0.3) in the high temperature region measured by means of the HR-MAS set-up. We started the measurement at 298 K and reached 366 K with steps of 2 K.

The most intense signal, which is cut off in the figure, belongs to hydration water protons and is more than four orders of magnitude larger than the other contributions. Note that, except for the peak at 1 ppm (assigned to the methyl functional group), all the peaks belonging to the protons of lysozyme, begin to appear at ≈ 320 K. This is a clear sign that the protons of lysozyme are immobile (protein side-chains are not flexible) on the NMR timescale at this hydration level up to ≈ 320 K. This temperature is on the border between the native state and the intermediate region where the unfolding process starts [26], [27].

The protein side-chains become more mobile on increasing the temperature, and their contribution to the free induction decay of the magnetization is indeed detectable. The temperature that marks the irreversibility of the unfolding process is ≈ 344 K where the magnetization signal suddenly increases and all protein contributions are clearly visible (Fig. 4). The decreasing of their peak width reflects the enhanced protein mobility due to the almost complete hydrogen bonding breakage.

## 4 Conclusions

In this paper we have studied the coupling of water with the hydrophilic moieties of hydrated lysozyme (h = 0.3) by means of Fourier Transform Infrared and NMR spectroscopy. In particular, by looking at selected vibrations we study how the hydrogen bond interaction governs the regime of protein activity. The intensity of the amide vibrational modes of hydrated lysozyme in the temperature interval 180 K < *T* < 350 K is able to reflect the thermal limit of biological functioning characterizing the studied system. Two temperatures, 225 K and 320 K, appear to be relevant for the onset of different dynamical regimes (and so to the functioning) of hydrated lysozyme. Their values agree with those already found for the same system and described in the introduction. Above 225 K the flexibility of hydrated lysozyme sharply increases due to the softening of the hydrogen bond network that the hydration water develops on its surface. Above 320 K water behaves as a normal liquid since the HB lifetime becomes too small (less than picoseconds). All water molecules become essentially free and water is no more a “good solvent”. The refolding rate constant assumes the maximum value [8] and lysozyme tends to lose its globular structure evolving toward a linear chain of amino acids.

Finally, in order to get precise insight into the thermal denaturation of hydrated lysozyme we followed the evolution of the proton NMR spectra for 298 K < *T* < 366 K with the High-Resolution Magic Angle Spinning probe. In the obtained spectra, all the peaks belonging to the protons of lysozyme, begin to appear at ≈ 320 K, except that at 1 ppm (assigned to the methyl functional group). This means that at this hydration level up to ≈ 320 K, the protein side-chains are not so flexible to be revealed by NMR. At higher temperatures, the protein side-chains become more mobile and their contribution is indeed well detectable. The temperature (≈ 344 K) at which the magnetization signal suddenly increases and all protein contributions are clearly visible signs the irreversibility of the unfolding process. The corresponding decrease of the peaks width reflects the increasing protein mobility provoked by the almost complete hydrogen bonding breakage.

In conclusion the experimental data we have presented in this work, suggest that the main mechanisms that define the thermal limits of the enzymatic activity of proteins are given by the hydrogen bond coupling between hydration water and protein hydrophilic groups.
